# Combination of Clinical and Gait Measures to Classify Fallers and Non-Fallers in Parkinson’s Disease

**DOI:** 10.3390/s23104651

**Published:** 2023-05-11

**Authors:** Hayslenne A. G. O. Araújo, Suhaila M. Smaili, Rosie Morris, Lisa Graham, Julia Das, Claire McDonald, Richard Walker, Samuel Stuart, Rodrigo Vitório

**Affiliations:** 1 Department of Sport, Exercise and Rehabilitation, Northumbria University, Newcastle upon Tyne NE1 8ST, UK; 2 Department of Physical Therapy, State University of Londrina, Londrina 86057-970, Brazil; 3 Northumbria Healthcare NHS Foundation Trust, North Tyneside General Hospital, Newcastle upon Tyne NE29 8NH, UK; 4 Gateshead Health NHS Foundation Trust, Gateshead NE8 2PJ, UK; 5 Department of Neurology, Oregon Health and Science University, Portland, OR 97239, USA

**Keywords:** Parkinson, gait, falls

## Abstract

Although the multifactorial nature of falls in Parkinson’s disease (PD) is well described, optimal assessment for the identification of fallers remains unclear. Thus, we aimed to identify clinical and objective gait measures that best discriminate fallers from non-fallers in PD, with suggestions of optimal cutoff scores. METHODS: Individuals with mild-to-moderate PD were classified as fallers (n = 31) or non-fallers (n = 96) based on the previous 12 months’ falls. Clinical measures (demographic, motor, cognitive and patient-reported outcomes) were assessed with standard scales/tests, and gait parameters were derived from wearable inertial sensors (Mobility Lab v2); participants walked overground, at a self-selected speed, for 2 min under single and dual-task walking conditions (maximum forward digit span). Receiver operating characteristic curve analysis identified measures (separately and in combination) that best discriminate fallers from non-fallers; we calculated the area under the curve (AUC) and identified optimal cutoff scores (i.e., point closest-to-(0,1) corner). RESULTS: Single gait and clinical measures that best classified fallers were foot strike angle (AUC = 0.728; cutoff = 14.07°) and the Falls Efficacy Scale International (FES-I; AUC = 0.716, cutoff = 25.5), respectively. Combinations of clinical + gait measures had higher AUCs than combinations of clinical-only or gait-only measures. The best performing combination included the FES-I score, New Freezing of Gait Questionnaire score, foot strike angle and trunk transverse range of motion (AUC = 0.85). CONCLUSION: Multiple clinical and gait aspects must be considered for the classification of fallers and non-fallers in PD.

## 1. Introduction

Falls are common in individuals with Parkinson’s disease (PD) and have devastating consequences [1]. Falling is twice as frequent in people with PD compared with those without the condition [1,2]. For those individuals who suffer falls, detrimental fall-related consequences include bone fractures, hospitalization and reduced mobility, impacting their quality of life [3,4]. Those aftermaths, combined with fear of falling, start a dysfunctional cycle and lead to even greater risk of falling [3]. Additionally, the consequences of falls represent high costs to patients and healthcare services [4]. Due to these serious consequences, falls figure as a public health concern [1,5]. In this context, it is necessary to devote attention to falls management in PD, including the identification of PD-specific markers for risk of falling [6,7].

Falls have a multifactorial etiology in PD and several risk factors have been identified [5,8,9,10]. Clinical aspects such as fall history, more severe/advanced disease, greater levodopa dosage, impaired postural control and cognitive deficits have been shown to increase the risk of falling in PD [8,11]. Additionally, gait deficits, such as increased variability, difficulties with dual-task walking and freezing of gait episodes, are among identified risk factors of falling in people with PD [1,7,12,13]. However, it remains unclear which of these risk factors best classify fallers and non-fallers among individuals with PD. Therefore, the direct comparison of multiple clinical and gait measures (and combinations of them) in classifying fallers and non-fallers in PD is of utmost importance.

The appropriate management of fall occurrence in PD requires the development of optimized assessment protocols for risk of falls. Standard clinical fall risk assessment in PD involves functional tests, balance scales, patient-reported symptoms and disease-specific clinical scales. Despite their easy applicability, these clinical tools have two major limitations: they are subjective and unable to measure subtle changes in gait. Therefore, fall risk assessment in PD may be enhanced by the inclusion of objective measures of gait. Recent studies demonstrate that the addition of objective measures of gait to clinical variables improved the classification of fallers and non-fallers in PD [6,14]. However, these studies have major limitations: the one by Delval et al. used an expensive motion capture system; Vitorio et al., despite applying low-cost wearable inertial sensors, assessed people with PD while OFF their medication (which limits ecological validity of findings). Furthermore, specific cut-off scores of objective measures of gait have not been proposed yet. Therefore, the current study was designed to address the above-mentioned limitations.

The primary aim of this study was to identify clinical (demographic, motor, cognitive and patient-reported) and objective gait measures that best discriminate fallers from non-fallers in PD, with suggestions of optimal cut-off scores. Additionally, we explored combinations of clinical and objective gait measures (i.e., clinical-only, gait-only and clinical + gait combinations) that best identify fallers in PD. We hypothesized that combinations of clinical and objective gait measures would better classify fallers and non-fallers among people with PD when compared to clinical-only or gait-only measures.

## 2. Materials and Methods

### 2.1. Participants

One hundred and twenty-seven individuals with mild-to-moderate PD [15,16] participated in this study. Participants were tested within 60 min of taking anti-Parkinsonian medication. Inclusion criteria were: (1) diagnosis of idiopathic PD according to UK Brain Bank criteria [17]; (2) aged 50 years or older; (3) independently able to walk; (4) stable medication for the month previous. Exclusion criteria included an inability to follow instructions to complete the study protocol, any other neurological disorders (other than PD) or musculoskeletal impairments that interfere with gait or balance.

The study was approved by the London-Bloomsbury NHS Research Ethics Committee (and Health Research Authority; 20/LO/1036, 5 October 2020) and the Institutional Review Board of the Oregon Health & Science University (#9903). All participants provided their written informed consent prior to the experiment. Assessments were carried out at the Balance Disorders Lab (Department of Neurology, Oregon Health & Science University) and the Clinical Gait Lab (Department of Sport, Exercise and Rehabilitation, Northumbria University).

### 2.2. History of Falls and Classification

Falls were defined as an unintentionally coming to the ground or some lower level not as a result of a major intrinsic event or an overwhelming hazard [6,11]. Based on self-reported history of falls, participants were classified as fallers (≥2 falls) or non-fallers.

### 2.3. Clinical and Gait Assessments

Participants underwent a clinical assessment, which included collection of socio-demographic information and medical history, clinical and cognitive tests. PD symptoms, severity and stage were assessed with the Movement Disorders Society (MDS-revised) Unified Parkinson Disease Rating Scale from Movement Disorders Society (MDS-UPDRS)—part III [15] and the Hoehn and Yahr rating scale (HY) [16], respectively. Global cognition was assessed with the Montreal Cognitive Assessment scale (MoCA) [18]; executive function was assessed by the Royall’s clock drawing [19] and Trail Making Test parts A and B [20]. Working memory was assessed through seated forward digit span. Visuospatial ability was measured by Benton’s Judgement of Line Orientation [21]. Fear of falling was assessed by the Falls Efficacy Scale—international version [22].

For the walking assessment, participants were instructed to walk, at a self-selected comfortable pace, back and forth on a straight 10 m walkway (tape marked at either end) for 2 min. Two walking conditions were tested: single and dual-task (the cognitive task was the maximum forward digit span) walking. Eight wearable inertial sensors (Opals, APDM Wearable Technologies—a Clario company, Portland, OR, USA) that included triaxial accelerometers, gyroscopes and magnetometers were used to instrument the walking tests. They recorded at 128 Hz and were attached, with Velcro straps, at the lumbar spine (5th lumbar vertebrae), sternum, bilaterally on the wrists, shins and feet. A total of 39 objective gait measures within 4 domains (upper/lower body, turning and variability) [23,24] were extracted using Mobility Lab software (Mobility Lab v2, APDM Wearable Technologies-a Clario company, Portland, OR, USA) [25,26,27]. Mobility Lab has been through test–retest reliability and validation testing; and outcomes can discriminate people with PD from healthy controls [23,25,26,27,28,29].

### 2.4. Statistical Analysis 

Data normality was assessed using the Shapiro–Wilk test. For demographic variables, comparisons between fallers and non-fallers were performed using the Mann–Whitney test or Student t test, according to data distribution. Receiver operating characteristic (ROC) curve analysis tested the performance of each outcome measure in discriminating fallers from non-fallers. The area under the curve (AUC), sensitivity and specificity were calculated. An AUC can be interpreted as follows; ≥0.9: outstanding, 0.8–0.9: excellent, 0.7–0.8: acceptable [30]. The optimal cut-off point of each outcome measure was determined as the point closest-to-(0,1) corner (false positive rate = 0%; sensitivity = 100%) in the ROC plane. 

*Combinations*. Only outcome measures with significant performance in classifying fallers and non-fallers were considered eligible for the combinations of outcome measures in different scenarios: clinical-only, gait-only (separately for single and dual-task walking) and clinical + gait measures. To avoid multicollinearity within the combinations, correlations matrices were built, and highly correlated outcome measures (r/rho ≥ 0.6) were excluded (keeping the one with the highest AUC for the combinations). Then, combinations of up to five outcome measures (due to our sample size) were considered for each of the above-mentioned scenarios. The optimal cut-off points observed for the individual measures were used for the combinations. For each hit cut-off score, one point was added to the final score of each combination. For example, if a participant reached the cut-off score in two individual outcome measures within the combination of five measures, the final score would be equal to two (out of five). Finally, ROC curve analysis was used to assess the performance of the combinations and the optimal cut-off score of each combination was determined (again as the point closest-to-(0,1) corner in the ROC plane) [31]. Statistical significance level was set at 0.05; all statistical analyses were performed using IBM SPSS version 27 (The International Business Machines Corporation, Armonk, NY, USA).

## 3. Results

### 3.1. Sample Characteristics

Thirty-one participants (24.4%) were classified as fallers (two or more falls) and 96 (75.6%) as non-fallers. Demographics and clinical characteristics are shown in Table 1. Fallers had more severe motor symptoms and more advanced disease stage than non-fallers (Table 1). 

### 3.2. Clinical Measures

Several clinical measures significantly classified fallers and non-fallers. Clinical measures with highest AUCs included: FES-I (AUC = 0.743, cut-off = 25.5 points), NFOGQ (AUC = 0.741, cut-off = 5.5 points), FOG status (AUC = 0.737, cut-off = 0.5), MDS-UPDRS III (AUC = 0.697, cut-off = 36.5 points), HY (AUC = 0.690, cut-off = 2.5) and TMT-B (AUC = 0.645, cut-off = 70.92 s). The performance parameters and cut-off scores of all clinical measures tested in the current study are presented as Supplementary Material (Table S1). 

The best three clinical-only combinations in classifying fallers and non-fallers had similar AUCs (0.808–0.809; Table 2). Out of these, the combination with the highest sensitivity (0.839) included the NFOGQ, MDS-UPDRS-III, HY and TMT-B; and the combination with the lowest false positive rate (0.208) had the same four measures plus FES-I (Table 2). 

### 3.3. Gait Measures during Single and Dual-Task Walking

**Figure 1:**
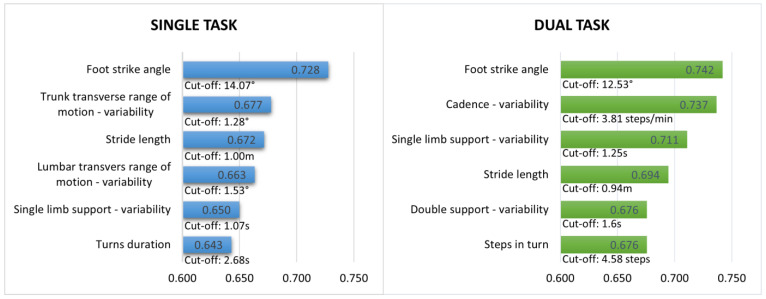
Gait measures (single and dual-task walking) with highest discriminative ability in classifying fallers and non-fallers.

Several gait measures recorded during single and dual-task walking had significant performance in classifying fallers and non-fallers (Figure 1). Interestingly, foot strike angle had the highest AUC for both single (AUC = 0728, cut-off = 14.07°) and dual-task walking (AUC = 0.742, cut-off = 12.53°). For single-task walking, other gait measures with high AUCs included: variability of trunk transverse range of motion, stride length, variability of lumbar transverse range of motion, variability of single limb support and turn duration. For dual-task walking, other gait measures with high AUCs included: cadence variability, variability of single limb support, stride length, variability of double support and steps in turn. The performance parameters and cut-off scores of all gait measures recorded in the current study are presented as Supplementary Material (Table S1). 

The best gait-only combinations for single and dual-task walking conditions had similar performance in classifying fallers and non-fallers. The best gait-only combination of single task walking measures included the foot strike angle, variability of trunk transverse range of motion, stride length, lumbar transverse range of motion variability and single limb support variability (AUC = 0.788, cut-off = 3.5 points; Table 3). The best gait-only combination for dual task walking included the foot strike angle, cadence variability, single limb support variability, stride length and double support variability (AUC = 0.790, cut-off = 2.5 points; Table 3).

### 3.4. Combination of Clinical and Gait Measures

**Figure 2:**
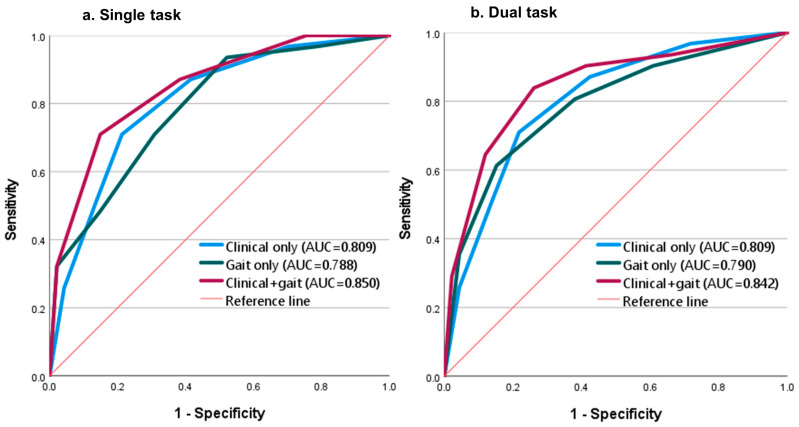
ROC curves of best combinations of clinical-only, gait-only and clinical + gait measures according to walking condition: (**a**) single task walking; (**b**) dual-task walking.

Overall, clinical + gait combinations performed better than clinical-only and gait-only combinations in classifying fallers and non-fallers (Figure 2). Considering only single-task walking measures, the best clinical + gait combination included: FES-I, NFOGQ, foot strike angle and trunk transverse range of motion variability (AUC = 0.850, cut-off = 2.5 points; Table 4). Considering dual-task walking measures, the best clinical + gait combination included: FES-I, foot strike angle, NFOGQ, cadence variability, and single limb support variability (AUC = 0.842, cut-off = 2.5 points; Table 4).

## 4. Discussion

This study tested the performance of clinical and objective gait measures, both separately and in combinations, to discriminate fallers from non-fallers in PD. Optimal cut-off scores were identified for all tested measures and combinations were built for different scenarios: clinical-only, gait-only (for both single and dual-task walking) and clinical + gait measures. Findings confirmed our hypothesis: the highest AUC in discriminating between fallers and non-fallers was achieved when combining clinical + gait measures. Our findings reinforce the well-described multifactorial nature of falls in PD [1,8,32,33] and have implications for the development of optimized fall risk assessment in PD, as discussed below.

No individual outcome measure had outstanding (AUC ≥ 0.9) or excellent (AUC = 0.8–0.9) discriminative ability in classifying fallers and non-fallers among people with PD. Top performing measures achieved only acceptable discriminative ability, namely FES-I (AUC = 0.743) and foot strike angle (AUC = 0.728/0.742, single/dual-task walking). Similar AUCs (<0.8) have been reported by both prospective (i.e., future falls) [34] and retrospective studies (i.e., classification of fallers and non-fallers) [6,35] testing the discriminative ability of individual clinical, functional and/or objective gait measures in PD. These findings highlight that a single measure is not enough for an accurate classification of risk of falls in PD. Further, the top-performing measures, FES-I and foot strike angle, indicate the association of specific impairments with falls in PD: (i) fear of falling has been frequently associated with occurrence of falls and reduced mobility and independence in people with PD [3,36,37]; (ii) reduced dorsiflexion while walking is a well-known characteristic of gait in PD, and has been shown to be affected by dual-task walking [38]. Therefore, it seems reasonable to suggest that both FEI-S and foot strike angle while walking should be considered for the development of fall risk assessment in PD. Moreover, fallers had more severe motor symptoms (MDS-UPDRS III) and more advanced disease stage (HY), highlighting that the risk of falls in PD increases with disease progression. 

The combination of clinical and gait outcomes better classified fallers and non-fallers among people with PD than clinical-only or gait-only combinations. Current findings are consistent with recent studies which demonstrated that the incorporation of objective measures of gait (quantified with wearable inertial sensors [6] or a motion capture system [14]) to standard clinical variables enhanced the classification of fallers and non-fallers in PD. Of note, the study by Vitorio and colleagues [6] assessed participants in the OFF levodopa state and had different gait measures in the top performing models: gait double support and turn duration variability. In the current study, gait measures that entered the top performing combination included foot strike angle and trunk transverse range of motion variability. These differences across the two studies suggest that the assessment of gait as part of fall risk assessment can be affected by PD medication state.

Specific gait measures, recorded using wearable inertial sensors, can enhance the traditional fall risk assessment in PD. Particularly, placement of inertial sensors on feet and trunk might be necessary for the classification of fallers and non-fallers as foot strike angle and trunk transverse range of motion variability had the highest AUCs among single walking measures. Further, our findings suggest that the dual-task condition used in this study (i.e., maximum forward digit span while walking) does not enhance the discriminative ability of combinations involving clinical and gait measures. Therefore, the forward digit span task while walking (not dual-task walking in general) may not be useful as part of fall risk assessment in PD as this would add time and complexity to the assessment. This is supported by previous research showing enhanced classification of falls in PD when a different dual-task condition (e.g., subtracting serial 7 s or 3 s) is considered [39,40].

Although the use of AUC as the primary measure is supported in the literature [31], the decision about the implementation of a model to classify fallers and non-fallers must also consider sensitivity and specificity. For example, the top two combinations of clinical + gait measures (single walk, Table 4) had three measures in common: FES-I, NFOGQ, and trunk transverse range of motion variability. The combination of these three measures correctly identified 83.9% of fallers and 75% of non-fallers (sensitivity and specificity, respectively); on the other hand, the combination involving these three measures and foot strike angle correctly identified 71% of fallers and 85.4% of non-fallers. A clear trade-off between sensitivity and specificity arises when foot strike angle is added to the combination involving FES-I, NFOGQ, and trunk transverse range of motion variability. This finding suggests that the foot strike angle adds more weight to the specificity.

The key strengths of the current study include the comprehensive clinical assessment (including motor and cognitive symptoms), use of objective and validated gait measures obtained with wearable inertial sensors, and the assessment in the ON state of PD medication. This approach covers the multifactorial nature of falls in PD and represents enhanced ecological validity in comparison to our previous study [6]. Particularly, wearable inertial sensors involve a low-cost, easy and quick setup that facilitate the use of gait assessment in clinical practice. Furthermore, because our sample included participants in stages 1 to 3 of PD, who were recruited from two centers in different countries, the generalization of findings is enhanced. However, the self-report of falls can be limited by subjective reporting. Moreover, considering that this was a retrospective study, current findings cannot be generalized to the prediction of future falls. Therefore, prospective studies are necessary to investigate whether the combinations of clinical and gait measures identified in the current study can predict future falls in PD. Current findings support that the use of technology may facilitate early identification of people with PD at risk of falling through enhanced accuracy of assessment protocols; technology can also facilitate early rehabilitation aiming to reduce the risk of falls [41].

## 5. Conclusions

Our findings suggest that combinations of clinical and gait measures have higher discriminative ability in classifying fallers from non-fallers among people with PD than combinations of clinical-only and gait-only measures. In particular, several combinations of clinical + gait measures had excellent discriminative ability (AUC > 0.8); combinations with the highest AUC included FES-I, NFOGQ, foot strike angle and trunk transverse range of motion variability. 

## Figures and Tables

**Table 1:** Sample characteristics.

Variables	All Participants (n = 127)	Fallers (n = 31)	Non-Fallers (N = 96)	*p*-Value
Age (years)	69.65 ± 7.67	70.73 ± 7.08	68.93 ± 8.00	0.06
Height (m)	1.68 ± 0.01	1.69 ± 0.09	1.68 ± 0.01	0.70
Weight (kg)	80.72 ± 17.56	77. 16 ± 14.54	83.08 ± 19.03	0.09
Education (years)	14.60 ± 3.40	14.64 ± 3.43	14.57 ± 3.41	0.79
Disease duration (years)	6.04 ± 5.23	7.18 ± 6.31	5.28 ± 4.26	0.13
MoCA (score)	26.98 ± 2.64	26.51 ± 2.87	27.29 ± 2.46	0.08
MDS-UPDRS III (score)	35.20 ± 16.17	39.42 ± 15.04	32.43 ± 16.40	0.02 *
HY (stage)	2.10 ± 0.65	2.27 ± 0.58	1.99 ± 0.68	0.03 *
1	20 (15.9%)	2 (6.5%)	18 (18.9%)	-
2	72 (57.1%)	16 (51.6%)	56 (58.9%)	-
3	34 (27.0%)	13 (41.9%)	21 (22.1%)	-

MoCA = Montreal Cognitive Assessment; MDS-UPDRS = Movement Disorder Society Unified Parkinson Disease Rating Scale; HY = Hoehn and Yahr scale; * *p* < 0.05.

**Table 2:** Best combinations of clinical measurements in classifying individuals with Parkinson disease as fallers or non-fallers.

Measures					AUC	Cutoff	Sensitivity	1−Specificity
1st	2nd	3rd	4th	5th				
FES-I	NFOGQ	UPDRS-III	HY	TMT B	0.809	2.5	0.710	0.208
FES-I	NFOGQ	HY	TMT B		0.809	1.5	0.806	0.344
NFOGQ	UPDRS-III	HY	TMT B		0.808	1.5	0.839	0.292
NFOGQ	HY	TMT B			0.802	1.5	0.677	0.188
FES-I	UPDRS-III	HY	TMT B		0.791	2.5	0.613	0.156

FES-I = International Falls Efficacy Scale; NFOGQ = New Freezing of Gait Questionnaire; UPDRS-III = Unified Parkinson Disease Rating Scale (part III); HY = Hoehn and Yahr scale; TMT B = Trail Making Test—part B.

**Table 3:** Best combinations of gait measurements (single and dual-task walking) in classifying fallers and non-fallers.

Measures					AUC	Cutoff	Sensitivity	1−Specificity
1st	2nd	3rd	4th	5th				
**Single task walking**								
Foot Strike Angle	Trunk Transverse ROM SD	Stride Length	Lumbar Transverse ROM SD	Single Limb Support SD	0.788	3.5	0.613	0.135
Trunk Transverse ROM SD	Stride Length	Lumbar Transverse ROM SD	Single Limb Support SD		0.787	2.5	0.613	0.177
Foot Strike Angle	Trunk Transverse ROM SD	Stride Length	Lumbar Transverse ROM SD		0.784	2.5	0.742	0.219
Foot Strike Angle	Stride Length	Lumbar Transverse ROM SD	Single Limb Support SD		0.779	2.5	0.645	0.208
Trunk Transverse ROM SD	Stride Length	Lumbar Transverse ROM SD			0.776	1.5	0.774	0.365
**Dual task walking**								
Foot Strike Angle	Cadence SD	Single Limb Support SD	Double Support SD		0.790	2.5	0.613	0.152
Foot Strike Angle	Cadence SD	Single Limb Support SD	Stride Length	Double Support SD	0.787	2.5	0.742	0.293
Foot Strike Angle	Cadence SD	Single Limb Support SD			0.783	1.5	0.774	0.293
Foot Strike Angle	Cadence SD	Single Limb Support SD	Stride Length		0.780	1.5	0.806	0.359
Cadence SD	Single Limb Support SD	Stride Length	Double Support SD		0.779	2.5	0.677	0.185

ROM = range of motion; SD = standard deviation.

**Table 4:** Best combinations of clinical + gait measures in classifying fallers and non-fallers.

Measures					AUC	Cutoff	Sensitivity	1−Specificity
1st	2nd	3rd	4th	5th				
**Single task walking**								
FES-I	NFOGQ	Foot Strike Angle	Trunk Transverse ROM SD		0.850	2.5	0.710	0.146
FES-I	NFOGQ	Trunk Transverse ROM SD			0.835	1.5	0.839	0.250
NFOGQ	Foot Strike Angle	Trunk Transverse ROM SD			0.831	1.5	0.774	0.250
FES-I	NFOGQ	Foot Strike Angle	UPDRS-III	Trunk Transverse ROM SD	0.828	2.5	0.806	0.240
FES-I	NFOGQ	Foot Strike Angle			0.817	1.5	0.806	0.240
**Dual task walking**								
FES-I	Foot Strike Angle	NFOGQ	Cadence SD	Single Limb Support SD	0.842	2.5	0.839	0.261
FES-I	Foot Strike Angle	NFOGQ	Single Limb Support SD		0.838	2.5	0.742	0.163
FES-I	Foot Strike Angle	NFOGQ	Cadence SD		0.837	2.5	0.742	0.141
FES-I	NFOGQ	Cadence SD	Single Limb Support SD		0.836	2.5	0.742	0.174
FES-I	NFOGQ	Single Limb Support SD			0.832	1.5	0.871	0.250

FES-I = International Falls Efficacy Scale; NFOGQ = New Questionary of Freezing of Gait; UPDRS-III = Unified Parkinson Disease Rating Scale (part III); ROM = range of motion; SD = standard deviation.

## Data Availability

All data are available from the corresponding author upon reasonable request.

## Supplementary Materials

The following supporting information can be downloaded at: https://www.mdpi.com/article/10.3390/s23104651/s1, Table S1: AUC, sensitivity, and 1-specificity values for all clinical and gait measures in classifying fallers and non-fallers among people with PD.
