# Supplementary material for: Combination of Clinical and Gait Measures to Classify Fallers and Non-Fallers in Parkinson’s Disease
Source: Sensors (Basel). 2023 May 11;23(10):4651. doi: 10.3390/s23104651 (PMC10221461; doi:10.3390/s23104651)
Supplement: Supplementary file 1 [file sensors-23-04651-s001.zip › sensors-2333399-supplementary.pdf]

SUPPLEMENTARY MATERIAL 1

Table S1-AUC, sensitivity, and 1-specificity values for all clinical and gait measures in classifying fallers and non-fallers among people with PD

Measure	AUC	Cut-off	Sensitivity	False positive rate
CLINICAL				
FES-I (points)	0.743	25.50	0.700	0.257
NFOGQ total	0.741	5.50	0.750	0.300
FOG status	0.737	0.50	0.800	0.343
MDS-UPDRS III total (points)	0.697	36.50	0.650	0.329
Hoehn & Yahr (stage)	0.690	2.50	0.350	0.129
TMT B (s)	0.645	70.92	0.750	0.329
TMT B-A (s)	0.642	37.51	0.700	0.314
Disease duration (years)	0.609	5.75	0.600	0.300
Education (years)	0.602	15.50	0.650	0.500
TMT A (s)	0.599	30.34	0.600	0.414
JLO (points)	0.597	24.50	0.550	0.357
CLOX1 (score)	0.566	13.50	0.400	0.257
CLOX2 (score)	0.562	13.50	0.600	0.557
MoCA (points)	0.556	27.50	0.550	0.417
Weight (kg)	0.553	77.21	0.500	0.361
Forward digit span (points)	0.530	6.50	0.750	0.653
Age (years)	0.528	67.50	0.650	0.571
Height (m)	0.509	1.71	0.650	0.500
SINGLE TASK WALKING				
Foot strike angle (°)	0.728	14.07	0.613	0.287
Trunk transverse ROM SD (°)	0.677	1.28	0.774	0.436
Stride length (m)	0.672	1.00	0.677	0.319
Lumbar transverse ROM SD (°)	0.663	1.54	0.774	0.436
Single limb support SD (s)	0.650	1.07	0.677	0.372
Turns duration (s)	0.643	2.68	0.677	0.372
Trunk coronal ROM SD (°)	0.632	0.79	0.613	0.436
Turn velocity (m/s)	0.619	97.47	0.581	0.330
Trunk transverse ROM (°)	0.615	6.23	0.677	0.426
Gait speed (m/s)	0.614	0.86	0.613	0.340
Trunk coronal ROM (°)	0.607	3.96	0.645	0.394
Double support SD (s)	0.605	1.43	0.677	0.479
Cadence SD (steps/s)	0.600	3.33	0.548	0.394
Double support (s)	0.598	24.10	0.548	0.298
Single limb support (s)	0.598	37.53	0.484	0.191
Step duration SD (s)	0.588	0.02	0.484	0.447
Cadence (steps/s)	0.585	104.71	0.677	0.436
Step duration (s)	0.584	0.57	0.677	0.426

Stride time (s)	0.584	1.15	0.677	0.436
Elevation midswing (°)	0.583	1.17	0.548	0.362
Lumbar coronal ROM (°)	0.577	5.54	0.613	0.457
Trunk sagittal ROM SD (°)	0.562	0.92	0.484	0.383
Stride time SD (s)	0.561	0.03	0.548	0.511
Arm ROM SD (°)	0.556	4.62	0.581	0.436
Elevation midswing SD (°)	0.550	0.43	0.710	0.500
Lumbar transverse ROM (°)	0.549	7.80	0.613	0.638
Lumbar sagittal ROM (°)	0.544	5.48	0.613	0.447
Arm ROM (°)	0.544	24.69	0.645	0.468
Steps in turn	0.543	1.15	0.484	0.277
Gait speed SD (m/s)	0.541	0.05	0.484	0.415
Lumbar sagittal ROM SD (°)	0.537	0.87	0.516	0.426
Trunk sagittal ROM (°)	0.533	4.09	0.581	0.362
Turns duration SD (s)	0.532	0.33	0.516	0.447
Arm swing velocity SD (m/s)	0.520	22.83	0.581	0.447
Turn velocity SD (m/s)	0.513	7.17	0.548	0.372
Arm swing velocity (m/s)	0.512	145.57	0.548	0.489
Lumbar coronal ROM SD (°)	0.510	0.65	0.452	0.415
Foot strike angle SD (°)	0.510	2.16	0.419	0.415
Stride length SD (m)	0.509	0.05	0.452	0.394
DUAL TASK WALKING				
Foot strike angle DT (°)	0.742	12.54	0.645	0.293
Cadence SD DT (steps/s)	0.737	3.81	0.742	0.337
Single limb support SD DT (s)	0.711	1.25	0.710	0.261
Stride length DT (m)	0.694	0.95	0.645	0.283
Double support SD DT (s)	0.676	1.60	0.645	0.304
Steps in turn DT	0.676	4.59	0.581	0.293
Trunk coronal ROM SD DT (°)	0.669	0.89	0.645	0.348
Stride time SD DT (s)	0.655	0.04	0.548	0.380
Trunk transverse ROM SD DT (°)	0.652	1.47	0.613	0.283
Step duration SD DT (s)	0.649	0.03	0.613	0.359
Turn velocity DT (m/s)	0.649	125.75	0.581	0.337
Gait speed DT (m/s)	0.641	0.80	0.581	0.326
Trunk coronal ROM DT (°)	0.629	3.83	0.645	0.391
Turns duration DT (s)	0.626	2.68	0.677	0.372
Trunk transverse ROM DT (°)	0.625	6.23	0.677	0.426
Double support DT (s)	0.624	23.05	0.774	0.435
Turns duration SD DT (s)	0.621	0.42	0.581	0.283
Single limb support DT (s)	0.621	38.05	0.645	0.348
Gait speed SD DT (m/s)	0.604	0.05	0.710	0.522
Stride length SD DT (m)	0.595	0.06	0.516	0.348
Cadence DT (steps/s)	0.538	103.49	0.613	0.446
Lumbar coronal ROM DT (°)	0.589	5.91	0.742	0.554
Lumbar transverse ROM SD DT (°)	0.579	1.64	0.548	0.326

Trunk sagittal ROM DT (°)	0.561	4.16	0.581	0.326
Elevation midswing DT (°)	0.559	1.16	0.613	0.391
Lumbar transverse ROM DT (°)	0.559	6.34	0.677	0.533
Arm ROM SD DT (°)	0.548	4.21	0.710	0.500
Lumbar sagittal ROM DT (°)	0.546	5.01	0.581	0.370
Trunk sagittal ROM SD DT (°)	0.544	0.98	0.548	0.370
Lumbar coronal ROM SD DT (°)	0.538	0.57	0.484	0.348
Step duration DT (s)	0.536	0.58	0.613	0.446
Stride time DT (s)	0.535	1.16	0.613	0.457
Elevation midswing SD DT (°)	0.532	0.33	0.710	0.696
Arm swing velocity DT (m/s)	0.525	147.52	0.581	0.489
Arm swing velocity SD DT (m/s)	0.518	22.38	0.452	0.326
Turn velocity SD DT (m/s)	0.514	12.81	0.548	0.446
Arm ROM DT (°)	0.513	23.87	0.484	0.467
Foot strike angle SD DT (°)	0.505	2.33	0.548	0.435
Lumbar sagittal ROM SD DT (°)	0.502	0.79	0.516	0.478

FES-I (International Falls Efficacy Scale); NFOGQ (New Freezing of gait Questionnaire); FOG (Freezing of gait); MDS-UPDRS III (Movement Disorders Society – Unified Parkinson Disease Rating Scale – part III); TMT (Trail Making Test), TMT A (TMT part A); TMT B (TMT part B); TMT B-A (difference between TMT parts A and B); JLO (Benton's Judgement of Line Orientation); MoCA (Montreal Cognitive Assessment); Kg (kilogram); s (seconds); m (meters); ° (degrees); ROM (range of motion); SD (standard deviation); DT (dual task)
